# From Compassionate Intuition to Clinical Competence: Spiritual Care Among Palliative Care Professionals in Ecuador

**DOI:** 10.3390/healthcare14193197

**Published:** 2026-09-28

**Authors:** Patricia Bonilla-Sierra, José Miguel Pérez-Jiménez, Rocío De Diego-Cordero

**Affiliations:** 1 Department of Health Sciences, Universidad Técnica Particular de Loja, Loja 1101608, Ecuador; 2 Inter-University Doctoral Program in Health Sciences, University of Seville, C/Avenzoar 9, 41009 Seville, Spain; 3 Human Resources and Research Unit, University Hospital Virgen Macarena, 41009 Sevilla, Spain; jpjimenez@us.es; 4 Department of Nursing, Schools of Nursing, Physiotherapy, and Podiatry, University of Sevilla, c/Avenzoar 6, 41009 Seville, Spain; rdediego2@us.es; 5 Research Group Comprehensive and Sustainable Health: A Bio-Psycho-Social, Cultural, and Spiritual Approach to Human Development—CTS-1149 (DesH-Global), 41009 Seville, Spain

**Keywords:** palliative care, spirituality, clinical competency, cultural competency, qualitative research

## Abstract

**Highlights:**

**What are the main findings?**

- Among the professionals interviewed, spiritual care was often supported by compassionate intuition, clinical sensitivity, and individual commitment, despite limited formal training, time constraints, and insufficient documentation tools.
- Participants described ethical tension related to family involvement, paternalism, intercultural communication, language barriers, and traditional or ancestral health practices.

**What are the implications of the main findings?**

- The findings point to possible areas for future development, including structured education in spiritual care, clearer documentation processes, and staff support strategies within palliative care settings.
- Future studies could further examine culturally sensitive mediation and collaboration with community or spiritual references as potential strategies to support respectful spiritual care in diverse contexts.

**Abstract:**

**Background/Objectives**: Spiritual care is essential for alleviating multidimensional suffering at the end of life. However, its clinical integration in Latin America faces cultural, linguistic, and religious complexities. These complexities also challenge the traditional biomedical model. This study addresses the gap between international theoretical frameworks and empirical reality in a diverse context by examining how healthcare professionals in Ecuador perceive, understand, and apply palliative spiritual care, using three competencies from the Latin American Association of Palliative Care (ALCP). **Methods**: This was a qualitative, exploratory, and descriptive study. We conducted in-depth, semi-structured interviews with 20 professionals (physicians, nurses, and psychologists) with experience in palliative care. We analyzed the data using inductive–deductive thematic analysis. **Results**: Spirituality was conceived as an existential dimension of purpose and transcendence. Clinical empathy drew upon the professional’s personal vulnerability and was channeled through nonverbal communication and informal interdisciplinary support. However, this approach faced critical institutional (time constraints and lack of clinical records), sociocultural (language barriers and clashes with traditional Andean medicine), and ethical–familial (paternalism and familism) barriers. **Conclusions**: Among the professionals interviewed, palliative spiritual care relied primarily on compassionate intuition, clinical sensitivity, and professional commitment, within a context marked by limited formal training and institutional, sociocultural, and ethical–familial barriers. These findings suggest possible areas for future development, including spiritual literacy, institutional self-care, and intercultural mediation, which should be examined in broader and more diverse contexts before being translated into practice or policy recommendations.

## 1. Introduction

Spiritual care is an essential dimension of comprehensive care for people with advanced chronic illnesses and those receiving palliative care. Since Cicely Saunders formulated the concept of “total pain,” end-of-life suffering has been understood as a multidimensional experience involving physical, psychological, social, and spiritual components [1,2]. Spiritual care, therefore, transcends religious accompaniment and is linked to the search for meaning, purpose, transcendence, and connection with oneself, others, nature, and what is significant or sacred.

This perspective, consistent with recent international literature and discussions from the VII International Congress on Science, Health, and Spirituality, supports spirituality as an evidence-based clinical and therapeutic resource within holistic health, particularly relevant in frailty, disease progression, and the approach of death [3,4,5]. The World Health Organization and international palliative care consensus have emphasized that relieving spiritual suffering is an ethical responsibility of health systems and care teams within person-centered care [6,7].

In this study, competence in spiritual care is understood, following the framework of the Latin American Association of Palliative Care (ALCP), as an integrated set of knowledge, skills, and attitudes that allows health professionals to identify, accompany, document, and respond ethically, compassionately, and with cultural sensitivity to the spiritual needs of patients in palliative care [4].

Evidence shows that spirituality and religiosity influence quality of life, coping, emotional distress, and symptom experience in patients with advanced diseases [8,9,10]. As an illustration of this pattern, in Latin American patients with advanced cancer, spiritual and religious beliefs were found to play an important role in coping; however, many patients reported unmet spiritual or religious needs, and spiritual pain was associated with greater physical suffering, anxiety, depression, and lower well-being [8].

Despite this recognition, the systematic integration of spiritual care into clinical practice remains limited. Recent evidence shows that professionals often report moderate competencies and insufficient training, even when they acknowledge the importance of spiritual care [11]. Perceptions of spirituality, prior training, clinical experience, religious affiliation, and educational level may influence professionals’ willingness and ability to provide this care [11,12,13]. However, most evidence comes from North America, Europe, or non-Latin American contexts, limiting its transferability to regions where spirituality is strongly shaped by relational, family, community, and intercultural values [8].

In Spain, recent research has highlighted spiritual care as a clinical and educational competency that remains insufficiently systematized. Evidence shows persistent barriers in medical practice, including a lack of training, time, and conceptual clarity [14], while nursing research emphasizes integrating theory, practice, self-awareness, and compassionate presence from the undergraduate level [15]. In home-based palliative care, studies have evaluated spiritual complexity along dimensions such as meaning, connection, and transcendence, reinforcing the need to incorporate it into interdisciplinary care routinely [16].

In Latin America, spiritual care is shaped by personalism, familism, and marked religious and cultural diversity, including Catholic, Evangelical, Indigenous, Afro-descendant, and syncretic traditions [4,8]. For example, families often make end-of-life decisions in the region rather than the patient on their own, reflecting the relational and familistic character of personalism.

This dynamic directly engages the ALCP competencies of ethics of spiritual care and empathetic, compassionate communication, as professionals must navigate collective decision-making while safeguarding the patient’s spiritual and religious needs. This context requires professionals to recognize spiritual needs beyond religious practices, accompany the patient without imposing beliefs, communicate with cultural sensitivity, and coordinate interdisciplinary responses to alleviate spiritual suffering [4,17]. However, evidence on how spiritual care competencies are perceived, interpreted, and applied in real Latin American clinical settings remains limited [4].

To address this gap, the Spirituality Commission of the Latin American Association for Palliative Care (ALCP) proposed a competency framework for quality spiritual care in Latin American palliative care, including six core competencies for interdisciplinary teams [4]. This framework provides a common language, observable standards, and a basis for professionalizing spiritual care.

These competencies are clinically relevant because they inform shared decision-making, empathic communication, and recognition of spiritual suffering as part of total pain. However, further research is needed to understand how these competencies are interpreted and applied in clinical practice, particularly where formal training is limited, and care often relies on intuition, personal experience, or individual sensitivity rather than institutionalized processes [18,19,20].

The Ecuadorian context offers a particularly relevant setting for this inquiry. Ecuador is characterized by a wide ethnic, cultural, linguistic, and religious diversity, as well as by the coexistence of biomedical models, ancestral medicine practices, and indigenous worldviews [21].

These elements justify studying spiritual care from a qualitative perspective, as it allows exploration of the meanings, tensions, and practices that emerge in clinical relationships. Qualitative research is especially relevant for understanding complex, subjective, and culturally situated experiences such as spirituality, suffering, compassion, and end-of-life decision-making [22]. Thus, analyzing professionals’ perceptions can help identify how they understand spiritual care, the barriers they face, the resources they use, and the training needs they consider essential for integrating it ethically, competently, and interculturally into palliative care [23].

This study analyzes how healthcare professionals in Ecuador understand and apply palliative spiritual care, based on three ALCP competencies: personal, spiritual, and professional development; ethics of spiritual care; and empathetic and compassionate communication.

## 2. Materials and Methods

### 2.1. Study Design

This qualitative, exploratory, descriptive study followed a hermeneutic–phenomenological interpretive orientation and used thematic analysis as the primary method for analyzing data. This approach suited the exploration of complex, subjective, and culturally situated phenomena in a profound, interpretive manner [24,25], such as suffering, compassion, and spiritual care at the end of life. In this study, the hermeneutic–phenomenological orientation informed the interpretation of participants’ narratives about meaning, suffering, compassion, and spiritual care in clinical practice. At the same time, thematic analysis provided the procedural strategy for coding, organizing, and interpreting the data. The study did not aim to develop a full phenomenological analysis of lived experience, but rather to understand how healthcare professionals in Ecuador perceive, give meaning to, and apply spiritual care in caring for individuals with advanced chronic illnesses and/or in palliative care. This design was appropriate given the phenomenon’s subjective, relational, and culturally situated nature, which involves experiences of meaning, hope, existential suffering, values, beliefs, and forms of clinical accompaniment [26].

Qualitative research is particularly well suited to studying spiritual care, as it allows exploration of complex phenomena that are difficult to measure or reduce to predetermined variables by capturing participants’ voices, experiences, and interpretations. In this field, semi-structured interviews offer a deep and flexible approach to understanding professionals’ experiences in clinical practice [26].

The study was based on the three competencies proposed by the Latin American Association of Palliative Care as its conceptual framework: personal, spiritual, and professional development; ethics of spiritual care; and empathetic and compassionate communication [4]. We selected these three competencies before data collection because they closely aligned with the research question and the study’s focus on professionals’ personal development, ethical decision-making, and empathic communication in spiritual care. Therefore, they directly informed both the interview guide and the deductive component of the analysis. The analysis followed an inductive–deductive logic: the competencies guided the initial organization of the data, while the emerging categories enabled the identification of barriers, facilitators, and training needs in participants’ discourse.

The design, conduct, analysis, and reporting of this qualitative study followed the Consolidated Criteria for Reporting Qualitative Research (COREQ), a 32-item checklist for transparent reporting of qualitative studies based on interviews and focus groups [27,28].

COREQ was selected because this study used in-depth semi-structured interviews to explore healthcare professionals’ perceptions and experiences of spiritual care. Its domains guided the description of the research team’s expertise and reflexive approach, study context, sampling strategy, interview guide development, expert validation, informed consent, interview procedures, audio recording, verbatim transcription, thematic analysis supported by ATLAS.ti 26, investigator triangulation, and anonymized quotations.

Applicable COREQ items are addressed throughout the Methods and Results sections. Non-applied procedures, such as transcript return and formal member checking, were reported transparently [27,28].

### 2.2. Study Context

The study was conducted in Ecuador, within the framework of care for people with advanced chronic illnesses and/or receiving palliative care.

Ecuador is a relevant setting for studying spiritual care because of its cultural, religious, linguistic, and territorial diversity, as well as the coexistence of biomedical models, community practices, ancestral medicine, and different forms of spiritual expression [21]. These characteristics make it necessary to understand how healthcare professionals interpret and apply spiritual care competencies in specific clinical contexts.

The study population comprised members of the Ecuadorian Association of Palliative Care (ASECUP). At the time of the study, ASECUP included 84 interdisciplinary members, including physicians, nurses, psychologists, social workers, priests, volunteers, and other professionals, which facilitated identifying informants with direct palliative care experience and clinical sensitivity to spiritual care [29].

The study used non-probabilistic purposive and convenience sampling to select participants with recognized experience in palliative care and potential exposure to spiritual care in clinical practice.

Eligible professionals were identified from the 84 ASECUP members and invited through the association’s institutional channels. Invitations were sent to physicians, nurses, psychologists, social workers, pastoral/spiritual care representatives, and volunteers with palliative care experience and potential exposure to spiritual care.

A total of 28 professionals were invited; 20 agreed to participate and completed the interview, while eight did not participate or declined before consent for personal, professional, or availability-related reasons.

Although the study sought heterogeneity in sex, profession, years of experience, work setting, sector, and level of care, the final sample reflected ASECUP’s membership profile and voluntary response to the invitation.

At the time of the study, ASECUP was predominantly composed of women and physicians, with most members working in urban settings: approximately 80% were women, 72% physicians, 9% psychologists, 6% nurses, and 13% other profiles, including social workers, pastoral/spiritual care representatives, volunteers, and biochemists. Although invitations included different professional profiles, only physicians, nurses, and psychologists responded and were therefore included in the final sample.

Some participants were professionally known to the research team through ASECUP or the palliative care field; however, no hierarchical, employment, or dependent relationship existed. Participation was voluntary, and no participant withdrew after providing consent.

The sample size was determined by data saturation, defined as the point at which interviews ceased to yield substantially new categories that addressed the study’s objectives [30,31,32]. This strategy is consistent with previous qualitative studies in spirituality and health, in which data collection continues until analytical sufficiency is reached. The two researchers responsible for coding assessed saturation jointly, based on the absence of substantially new codes or themes and using the same iterative, reflexive consensus process used for category refinement, as described in Section 2.5. Saturation was reached during the final interviews, when no substantially new codes or themes emerged.

### 2.3. Inclusion and Exclusion Criteria

The inclusion criteria were as follows:

- Healthcare professionals aged 18 or older with experience in the care of people with advanced chronic illnesses and/or palliative care.
- Voluntary agreement to participate and signature on the informed consent form.
- Availability to participate in a semi-structured, virtual interview and authorization for recording for analysis purposes.

The exclusion criteria were as follows:

- Professionals without direct experience in palliative care or the care of people with advanced chronic illnesses.
- Exclusively administrative staff.
- Religious leaders or pastoral agents who were not part of a healthcare team.
- Participants who did not complete the informed consent form or decided to withdraw from the study.

### 2.4. Data Collection Technique and Instrument

Data were collected through in-depth, semi-structured virtual interviews, which allowed exploration of participants’ experiences, perceptions, knowledge, and professional practices while maintaining a common structure and the flexibility to address emerging issues [33]. Interviews were conducted individually via Zoom in a private, quiet place chosen by each participant, with only the interviewer and the participant present. Interviews lasted approximately 45–60 min. Each participant completed one interview, except in one case, where the interview was repeated because the first session was not successfully recorded; no repeat interviews were conducted for analytical purposes.

The interview guide was developed based on the study objectives and the conceptual framework of competencies for quality spiritual care proposed by the Latin American Association of Palliative Care (ALCP). It included a section on sociodemographic and professional data, followed by open-ended questions organized around three competencies: personal, spiritual, and professional development; ethics of spiritual care; and empathetic and compassionate communication. The questions explored professional training, palliative care experience, spirituality training, understanding of spiritual care, application of ALCP competencies, barriers, facilitators, training needs, and opportunities for improvement (Table 1).

Seven experts in palliative care, spirituality, bioethics, clinical care, and qualitative research validated the interview script. They assessed the clarity, coherence, relevance, and sufficiency of the questions according to the study objectives and selected competencies [34]. This expert review, consistent with previous qualitative studies on spiritual care [35], allowed refinement of wording, reduced ambiguity, improved thematic sequence, and strengthened content validity and methodological rigor.

Researchers with experience in palliative care, spirituality, and qualitative research conducted the interviews virtually to ensure privacy and confidentiality. Before each interview, participants received information about the study’s purpose, voluntary participation, their right not to answer, and the option to withdraw without consequences. With prior authorization, the researchers audio-recorded and transcribed interviews verbatim to preserve the narrative and emotional richness required for rigorous qualitative analysis [26].

### 2.5. Data Analysis

The analysis was conducted based on verbatim interview transcripts. The researchers first conducted repeated readings to become familiar with the data and identify relevant units of meaning (familiarization phase). The researchers then used open coding during the initial coding phase to identify expressions, concepts, and patterns related to perceptions and application of spiritual care competencies. Coding focused on meaning units and thematic segments rather than isolated words, allowing participants’ narratives to be interpreted within their clinical and contextual meaning.

The analysis followed a hybrid deductive-inductive approach. Deductively, the three selected ALCP competencies guided the initial organization of the data. Inductively, the analysis identified emerging categories and subcategories from participants’ experiences (theme generation). The team iteratively reviewed, compared, and refined categories to ensure coherence among codes, categories, and meanings (reviewing and defining themes) [36].

ATLAS.ti 26 was used to organize transcripts, codes, and text segments, supporting the traceability of the analytical process without replacing the researchers’ interpretive judgment. Two researchers coded and interpreted themes. The team discussed emerging codes, categories, and potential interpretive biases until they reached agreement, strengthening coherence among the data, analytical categories, and final themes. While general interview questions explored barriers, facilitators, and training needs, participants’ narratives shaped the specific configuration of barriers. Descriptive codes, such as time constraints, lack of documentation tools, language barriers, traditional medicine, confusion between spirituality and religiosity, death-related taboos, family control, and dogmatic tensions, were grouped into institutional, sociocultural, and ethico-familial barriers.

To further strengthen analytical transparency, Supplementary Table S1 provides representative examples of the audit trail from anonymized meaning units to initial codes, analytical categories, and final themes.

The interpretive construct of “compassionate intuition” was not treated as a predefined ALCP competency, a direct participant label, or an initial code. Instead, we developed it as an author-generated higher-order interpretive construct grounded in recurrent patterns across the dataset. These patterns included personal vulnerability as a source of empathy, nonverbal presence, ethical-family mediation, informal team support, and limited formal training or documentation. As shown in Supplementary Table S1, we first identified these patterns as meaning units and initial codes, then grouped them into analytical categories and final themes, and finally integrated them into the interpretive construct of “compassionate intuition”.

### 2.6. Methodological Rigor

Methodological rigor was strengthened in line with the COREQ domains, with attention to credibility, dependability, confirmability, transferability, and reflexivity [27,28]. Credibility was supported through private semi-structured interviews, audio recording, verbatim transcription, repeated reading of transcripts, and anonymized quotations. Dependability and confirmability were enhanced through an expert-validated interview guide, coding by two researchers, iterative category review, triangulation across the research team, and reflexive discussion of analytical decisions.

To ensure consistency, the research team conducted an internal audit of the anonymized dataset, reviewing quoted excerpts against participant codes, sociodemographic attributes, analytical categories, and corresponding ALCP competencies or inductive themes. Triangulation was limited to investigator triangulation, as the study used a single-source interview design and one conceptual framework. We supported transferability by describing the Ecuadorian context, ASECUP-based recruitment, participant characteristics, and care settings.

Regarding reflexivity, some participants were professionally known to members of the research team through ASECUP. This prior professional familiarity may have influenced the interview dynamic or how the researchers interpreted participants’ accounts. To reduce this risk, the researchers used a standardized, expert-validated interview guide, anonymized transcripts, coding by two researchers, and reflexive discussion of emerging codes and interpretations until they reached consensus. The absence of transcript return, formal member checking, and field notes may have limited direct participant validation and the documentation of contextual impressions during data collection. Therefore, credibility was supported mainly through verbatim transcription, repeated transcript review, investigator triangulation, an internal audit of the participant–quotation matrix, and reflexive discussion of analytical decisions.

### 2.7. Ethical Considerations

The Ethics Committee for Research in Human Beings of the Universidad Técnica Particular de Loja (CEISH-UTPL) approved the study, with approval code 2025-07-INT-EO-RM-012. All participants signed an informed consent form, and the study guaranteed confidentiality, anonymization, and exclusive scientific use of the information collected during the interview. The audio recordings, transcripts, consent forms, and coding files were stored in password-protected digital folders accessible only to the research team; consent forms were stored separately from the interview data, and the analytical dataset contained only anonymized transcripts and coded excerpts.

### 2.8. Use of Generative Artificial Intelligence

Artificial intelligence (AI) tools were used exclusively to support grammar, language refinement, and manuscript readability, and the visual design of Figure A1, under strict human supervision. ChatGPT-4 was used only for grammar, language refinement, and readability support, while Nano Banana Pro was used only to assist with the visual design of Figure A1. No AI tool generated analytical content, codes, categories, themes, results, or interpretation. The authors have thoroughly reviewed and edited all AI-assisted outputs and assume full responsibility for the accuracy, integrity, and final content.

## 3. Results

### 3.1. Participants

#### Sociodemographic Characteristics of the Sample

The sample consisted of 20 healthcare professionals: 14 physicians, 4 nurses, and 2 psychologists. Ninety percent were women and 10% were men. Participants had diverse professional backgrounds, with a mean of 45.6 years (range: 30–72 years) and an average of 21.1 years of clinical experience.

Regarding place of residence, participants were primarily located in Quito (55%), followed by Loja (20%), Guayaquil (15%), Machala (5%), and Riobamba (5%). In terms of employment, 70% of the professionals worked in the public sector and 30% in the private sector. Regarding the level of care provided, 55% worked at the secondary level, 25% at the primary level, and 20% at the tertiary level.

Regarding academic qualifications, 90% of participants held postgraduate degrees in medical specialties or multidisciplinary master’s degrees in palliative care. Additionally, 10% had a diploma in palliative care, that is, specific supplementary training in this area. Regarding spirituality, only 10% reported formal training through a diploma or other structured studies, while 90% reported no specific training in this area.

Table 2 presents the sample’s sociodemographic characteristics.

Before presenting the narrative findings, Table 3 provides an overview of the thematic structure derived from the analysis, including the main themes, subthemes, and representative quotations. This table guides readers through the organization of the Results section.

### 3.2. Competencies of Spiritual Care in Clinical Practice

Analysis of the interview data identified three core competencies that structure spiritual care in clinical practice: personal, spiritual, and professional development; ethics of spiritual care; and empathetic and compassionate communication. The first includes spiritual self-concept, personal growth, emotional exhaustion, self-care, and team training. The second addresses self-care as an ethical imperative, person-centered care, and end-of-life ethical challenges. The third focuses on empathic communication, documentation of spirituality, sociocultural and religious barriers, and interdisciplinary work. Together, these categories reflect a comprehensive model of spiritual care shaped by personal, clinical, and contextual factors.

#### 3.2.1. Personal, Spiritual, and Professional Development

##### Self-Concept and Understanding of One’s Own Spirituality

The professionals interviewed in this study described spirituality as a process of self-awareness and personal development that helped them recognize and manage their fears, preventing them from interfering with patient care. For these participants, spiritual care meant searching for purpose, connecting to their own life story, and transcending strictly religious frameworks.

This holistic perspective was clearly evident in their narratives, in which they identified spirituality as the driving force of human existence: “I believe spirituality is what gives meaning to a person” (C1, female, 34, physician). In the end-of-life context, the professionals understood this dimension as a process of integration and reconciliation: “For me, spirituality goes beyond religion… it’s like a search for meaning… a purpose in their illness and in this process of integrating death as a part of life” (C20, female, 41, physician).

Likewise, the professional’s own spirituality acted as a support in the face of adversity: “it is that dynamic part that makes me look for the meaning, the objective of my existence… my suffering has transcended so much that I think I can tell you that everything that has happened has meaning” (C15, female, 53, physician).

Participants also linked spirituality with personal purpose, life coherence, and its integration into daily practice: “spirituality is the transcendence I have… as a person in what I was, in what I am, and in what I intend to be” (C3, female, 58, physician); “finding meaning in what I do, always valuing my priorities” (C4, female, 33, physician); “something transversal in all the acts of my life” (C14, female, 37, physician).

Although several professionals reported a religious background, primarily Catholic, they showed conceptual flexibility by differentiating clinical spirituality from religiosity and adapting to the patient’s beliefs. This openness was expressed in the coexistence of personal practices (such as prayer) with an inclusive attitude toward other worldviews: “I am Catholic… prayer is a self-help tool” (C8, female, 30, psychologist); “there will always be a connection with a higher power… I have approached my life from that perspective” (C6, female, 41, physician).

Taken together, these findings show that these professionals’ spiritual self-concept constitutes a fundamental clinical resource that fosters more empathetic, respectful, and person-centered care.

##### Impact of Personal Growth on Compassionate Care

Personal experiences of vulnerability were key to building deep empathy among these professionals, bridging their understanding of patient suffering. Situations such as serious illness or the loss of loved ones transformed the clinical perspective, fostering a more humane and sensitive approach: “It allowed me to better understand what the people I care for feel” (C7, female, 60, physician); “It helped me explore a little more about what is important to patients” (C13, female, 38, physician).

Likewise, life roles, especially motherhood or fatherhood, strengthened the capacity for emotional connection, broadening the understanding of pain from a relational and affective perspective: “one learns to understand a little more… the care of the person is suffering” (C9, female, 38, physician).

The findings suggest that these professionals perceived their personal growth and significant experiences as shaping their spiritual dimension and capacity to provide more compassionate, empathetic, and person-centered clinical support.

##### Emotional Burnout and Self-Care Strategies

The interviewed professionals recognized burnout and compassion fatigue as inherent risks in palliative care, stemming from both emotional over-involvement with patients, especially when there is personal identification, and institutional limitations: “that involves us more than necessary and could end in emotional burnout” (C4, female, 33, physician); “the daily struggle… with the administrative side” (C3, female, 58, physician).

In response, they developed primarily intuitive and unstructured self-care strategies focused on emotional self-regulation and setting personal boundaries. These included reflective, recreational, and disconnection practices: “writing… as a self-regulation tool” (C8, female, 30, psychologist); “recognizing my limits” (C3, female, 58, physician); “going for walks… exploring mountains” (C17, female, 39, physician).

Self-care emerged as a fundamental resource for sustaining clinical practice, although its implementation depended more on individual initiatives than on formalized institutional programs.

##### Training, Continuing Education, and Team Support

The results showed that, in the absence of formal structures, the interdisciplinary team constituted the main emotional and operational support for the professionals, functioning as a space of containment and accompaniment: “If it weren’t for the team, where would I be?… my colleagues help me” (C16, female, 52, nurse); “a good palliative care specialist has to have… a good psychologist… who helps you with tools to be able to vent” (C5, male, 47, physician).

However, a significant gap was identified in theoretical and structured training in spirituality, which led to practices based on intuition and experience rather than formalized competencies: “training is extremely important… so as not to improvise” (C4, female, 33, physician); “there is a huge gap in knowledge of spirituality” (C3, female, 58, physician); “we need more guidelines… to be able to provide help” (C5, male, 47, physician).

The findings show a strong valuation of spiritual care and self-care as ethical and clinical pillars, but also a reliance on empirical learning and informal support. These patterns suggest possible areas for future development, including formal education, institutional guidance, and structured support for professionals.

#### 3.2.2. Ethical Competencies of Spiritual Care

The analysis revealed that, among the professionals interviewed, the ethics of spiritual care centered on three core axes: self-care as an ethical duty, the centrality of the patient’s dignity and autonomy, and managing complex end-of-life dilemmas.

First, self-care is understood not only as a wellness strategy but also as an ethical-clinical imperative, essential for preventing depersonalization and ensuring compassionate care: “*If I am not well, how can I help my patient?*” (C16, female, 52, nurse).

Second, the professionals placed the patient at the center of care, prioritizing respect for their life story, values, and decisions through a non-judgmental, person-centered approach: “*The patient is the protagonist, and their autonomy must be respected*” (C4, female, 33, physician).

Finally, significant ethical challenges emerged, especially when patient autonomy conflicted with family decisions, religious beliefs, or sociocultural constraints. These tensions could compromise care quality and cause avoidable suffering, forcing the professional to act as an ethical mediator. In this scenario, bioethics committees became a key resource for complex decision-making: “*We had to go to the ethics committee… who supported us*” (C16, female, 52, nurse).

The findings show that, among the professionals interviewed, ethical spiritual care is characterized by a strong humanistic and person-centered orientation. However, participants also described challenges related to family dynamics, cultural diversity, and institutional limitations, suggesting a need for structured approaches and interdisciplinary support in the settings represented in this study.

#### 3.2.3. Empathic and Compassionate Communication in Spiritual Care

Participants described empathic and compassionate communication as a relational practice centered on presence, active listening, emotional sensitivity, and careful adaptation of information to the patient’s needs. These professionals emphasized the importance of “being with” the patient, using silence, proximity, eye contact, and nonverbal gestures as part of spiritual accompaniment.

This was reflected in narratives where these professionals highlighted the importance of sitting with the patient, asking for permission to speak, and adapting the conversation to the patient’s readiness: “*The most important thing is the face-to-face interaction… sitting down and asking permission to speak*” (C11, female, 43, physician). In situations of high vulnerability, communication was sometimes sustained mainly through presence: “*We came to communicate only with this [holding hands]… communication was presence*” (C8, female, 30, psychologist).

Participants also described the need to communicate truth gradually and compassionately, adapting information to the patient’s emotional process without imposing professional views. One participant referred to this as “*the tolerable truth for the patient*” (C17, female, 39, physician).

Interdisciplinary teamwork facilitated spiritual communication in complex situations. Participants reported that they often shared information informally during rounds, hallway conversations, or shift changes, which they perceived as helpful for coordinating responses to patients’ emotional and spiritual needs.

In some cases, this coordination enabled meaningful actions, such as facilitating rituals, farewells, or family encounters, which participants perceived as part of spiritual accompaniment.

Overall, participants perceived empathic and compassionate communication as a relational competency based on presence, active listening, emotional sensitivity, and team coordination. Section 3.3 presents cross-cutting barriers that limit this communication.

### 3.3. Barriers

#### 3.3.1. Institutional and Structural Barriers

Participants identified time constraints, care pressure, administrative burden, and the absence of documentation tools as the main institutional barriers to spiritual care. In public hospitals, productivity demands were perceived as limiting the time available for existential or spiritual conversations: “*In public hospitals […] what matters is production […] they systematize us a lot and don’t allow us time, and for us to have those spiritual connections we need to have time*” (C19, male, 52, physician).

Participants also described administrative tasks as a source of exhaustion that reduced the time and attention available for holistic care: “*more than attending to the patient itself, the administrative tasks are exhausting*” (C20, female, 41, physician). In addition, they reported the absence of specific formats or guidelines for documenting spiritual care in clinical records, which made this dimension difficult to integrate into care planning and interdisciplinary follow-up.

Finally, participants noted limited structural support for formalizing spiritual care roles, alliances, or referral pathways within the health system. As one participant stated, there were “*limitations in funding, budgets, and job classifications*” for integrating spiritual care specialists into healthcare teams (C19, male, 52, physician).

#### 3.3.2. Sociocultural and Educational Barriers

Participants described Ecuador’s sociocultural diversity as a source of communication challenges in spiritual care. Differences in educational level and health literacy were perceived as making conversations more complex, particularly when patients had limited reading or writing skills: “It’s not the same talking to someone who has at least finished school as talking to someone who can’t read and write… the conversation is much more complex” (C20, female, 41, physician).

Participants also reported tensions between biomedical care and traditional or ancestral health practices. One participant stated, “the patient believes in ‘cleansers’ in shamans” (C20, female, 41, physician), which required professionals to approach these beliefs with respect and without imposing biomedical views.

Finally, participants identified conceptual confusion between spirituality and religiosity, as well as fear and stigma surrounding death, as barriers to open dialogue about spiritual needs and end-of-life care. As one participant stated, “they confuse spirituality with religiosity, so that’s also a limitation” (C1, female, 34, physician). Another participant described death as a persistent taboo: “The main one is that taboo they have about death… they are very afraid of it” (C15, female, 53, physician).

#### 3.3.3. Ethical and Family Barriers (Paternalism and Dogma)

Participants described family dynamics as a recurrent source of ethical tension in spiritual care, particularly when relatives attempted to influence information disclosure, decision-making, or the patient’s expression of preferences. These situations were perceived as challenging because professionals had to balance family involvement with respect for the patient’s dignity, autonomy, values, and spiritual needs.

One participant described situations in which “family members don’t want the patient’s decision respected; they want things done their way” (C1, female, 34, physician). Another participant described cases in which relatives limited the patient’s ability to speak openly, “when they don’t let them express their thoughts” (C2, female, 61, nurse). Participants also reported that strong religious or dogmatic positions within the family could hinder clinical and spiritual accompaniment, as illustrated by the following account: “the mother practically wouldn’t let us near them or do anything” (C12, female, 72, physician).

These accounts suggest that, for participants, ethical spiritual care often required mediation between patients, families, beliefs, and clinical decisions, especially in end-of-life contexts.

## 4. Discussion

### 4.1. Competency 1: Personal, Spiritual, and Professional Development

Personal, spiritual, and professional development is a fundamental pillar of compassionate palliative care and requires self-awareness and critical reflection. In this study, clinicians’ own spirituality and recognition of personal vulnerability emerged as catalysts for empathy and spiritual humility [37]. This aligns with the literature, which shows that spiritual competence depends on intrapersonal resources, with inner growth serving as a therapeutic tool for person-centered care [4,38]. Consciously integrating spirituality into care also supports deeper clinical connection and benefits the well-being of both patients and the interdisciplinary team [39].

From this perspective, spiritual care fits within the broader humanizing healthcare framework as comprehensive, compassionate, patient-centered care that addresses the biological, psychological, social, and spiritual dimensions of the sick person [40]. Denying this dimension in clinical practice can contribute to dehumanization, as SECPAL warned in its 2011 manifesto [41]. However, persistent barriers limit its incorporation, including lack of time, absence of protocols, fear of intruding on the patient’s privacy, and insufficient specific training. Therefore, authors such as Koenig [42] and Puchalski [43], along with recent approaches that recognize spirituality as a determinant of health, have defended the need to integrate spiritual care into health training, clinical practice, and health systems, not as an accessory skill, but as a competence linked to quality, resilience, and comprehensive care [44].

The international literature underscores that self-awareness and clinical reflection are essential mechanisms for preventing compassion fatigue and burnout. In end-of-life care, reflective capacity helps professionals establish healthy emotional boundaries and prevent their own fears or unresolved suffering from interfering with patient care. This self-awareness supports self-compassion, a key mediator in reducing depersonalization, promoting compassion satisfaction, and strengthening professional resilience [45,46]. Therefore, holistic care requires cultural and institutional changes that integrate spiritual needs assessment models and empower both professionals and patients in high-demand clinical settings [47].

Despite their proven importance, implementing these preventive measures faces serious structural limitations. In line with recent research on palliative care professionals in Ecuador [48], our qualitative data reveal a pattern of insufficient self-care practices among the professionals interviewed. Professionals tend to rationalize this deficiency by citing extreme pressure from patient care, time constraints, and heavy administrative burdens, which leads them to adopt short-term, reactive coping strategies. Consequently, emotional support often falls almost exclusively on informal support networks among colleagues. While individual initiative and self-care cannot rely solely on a worker’s will, these findings suggest that healthcare institutions could support professionals by promoting policies and structured spaces for self-reflection and team development [48].

This need for institutional support has also been evidenced in Ecuadorian nurses, who presented moderate levels of coping with death despite their frequent exposure to end-of-life patients. Although many professionals reported some willingness to talk about death, qualitative findings revealed emotional distress, fear of suffering, difficulties communicating with patients and families, and a need for more training in palliative care, emotional support, and spiritual accompaniment [49].

Finally, the data suggest a strong reliance on relational intuition in contexts with limited formal education. These findings point to a possible area for future educational development: strengthening sensitivity and spiritual literacy within health-science curricula, particularly in contexts where spiritual care remains largely experiential and insufficiently formalized [50].

Conceptually, “compassionate intuition” is related to clinical intuition, compassionate presence, relational competence, spiritual sensitivity, and experiential knowledge, but it is not equivalent to them [4,15,18,19,20,50]. In this study, it refers to an unstructured, self-taught, relational, and ethical way of responding to spiritual suffering through empathy, nonverbal presence, ethical-family mediation, informal team support, and culturally sensitive responses. Thus, it describes a preliminary experiential mode of care that emerged in contexts of limited formal training, documentation tools, and institutional protocols, rather than an established professional competency.

Our findings extend previous research by showing that, among the professionals interviewed, spiritual care is not absent from clinical practice; rather, it is often practiced through compassionate intuition, informal team support, and personal clinical sensitivity, despite limited formal training. This reliance on relational intuition reflects a gap between the value attributed to spiritual care and the availability of structured educational and institutional support. According to the ALCP, generalist spiritual care includes basic competencies for all team members, such as compassionate listening, screening, and spiritual history-taking. In contrast, specialized care requires trained professionals to address complex spiritual suffering, belief conflicts, or profound existential dilemmas [4].

Integrating these concepts into health professional education may also foster reflection on personal beliefs, diversity, and inclusion, promoting an ethical approach sensitive to the region’s sociocultural complexity [51].

### 4.2. Competence 2: Ethics of Spiritual Care

The ethical dimension of spiritual care, as understood by the participants in this study, aligns with the international literature that reframes self-care not as an optional resource but as a professional and institutional obligation. The finding that the professionals interviewed perceived their own well-being as a precondition for compassionate care aligns with evidence showing that burnout and compassion fatigue may compromise patient safety and the quality of clinical care [52].

Other authors have shown that self-compassion mediates the relationship between professionals’ inner lives and outcomes such as burnout and compassion fatigue, supporting the need to integrate self-care into institutional structures rather than leaving it solely to individual discretion [45]. In this study, participants described self-care practices as mostly intuitive and unstructured, with limited formal institutional support. This may reflect a structural vulnerability also reported in other low- and middle-income health systems [53] and suggests a need to further examine context-sensitive policy responses in Ecuador.

Regarding person-centered care and respect for patient autonomy, the strong humanistic orientation among participants aligns with the foundational bioethical principles established by Beauchamp and Childress [54] and with international palliative care standards that regard failure to elicit patient preferences as a violation of both autonomy and dignity. However, a distinctive feature of the Ecuadorian context and one that goes beyond what the results alone describe is the tension between an individually framed conception of autonomy and the deeply relational decision-making structures prevalent in Latin American cultures.

Familismo, widely described in the Latin American palliative care literature, positions the family as a legitimate moral actor rather than an obstacle to autonomy [55]. Within the settings represented in this study, this relational view was especially relevant because participants described decision-making as frequently shaped by family and community networks.

These findings suggest that end-of-life decision-making may be better understood as an interdependent process shaped by affective and cultural ties. A relational autonomy approach may help clinicians respect the patient’s voice while acknowledging the collective nature of decision-making in this context [56].

The end-of-life ethical dilemmas described by participants, especially tensions between patient autonomy, family wishes, and religious beliefs, are not unique to Ecuador. However, the country’s sociocultural diversity and the coexistence of Indigenous worldviews with Western biomedical models intensify them. Similar cultural tensions have been associated with moral distress among professionals working within biomedical frameworks [57]. Participants’ reference to bioethics committees as a support mechanism is consistent with evidence that ethics consultations help resolve conflicts between families and clinical teams [56]. Therefore, these findings suggest strengthening ethical support structures, particularly at the care levels represented by participants.

Taken together, these findings show that the ethics of spiritual care cannot rely solely on individual commitment. It requires systemic support that addresses professionals’ well-being, the cultural complexity of decision-making, and institutional mechanisms for end-of-life ethical deliberation.

### 4.3. Competence in Empathic and Compassionate Communication

The analysis of empathic and compassionate communication [4] shows that the palliative care professionals interviewed in this study prioritized presence over words, using active listening, silence, and physical contact as key forms of spiritual support. This reflects the value of personalism and suggests that, in the face of existential suffering, genuine presence can function as a therapeutic intervention. As Taylor [58] notes, words may be insufficient in profound spiritual distress; therefore, nonverbal communication and adapting to the patient’s pace are essential to avoid directive responses and support patient self-awareness.

However, this clinical sensitivity contrasts with the limited documentation of spiritual care. Spiritual needs often appear absent from electronic health records, are recorded only partially, or are shared informally among professionals. This creates an ethical tension between ensuring the continuity of interdisciplinary care and protecting the confidentiality of intimate, spiritual, and existential information. Among the professionals interviewed, the absence of standardized documentation and fear of disclosing sensitive information were perceived as factors that contributed to compassionate but informal spiritual care, limiting its continuity in line with ALCP standards [4,59].

A particularly relevant finding is that, among the professionals interviewed, language barriers, traditional medicine, and Indigenous worldviews were perceived as shaping empathic communication. Rather than representing Ecuadorian healthcare as a whole, this finding reflects experiences reported within the settings of this study. It is consistent with the previous literature describing how, in some Ecuadorian Indigenous and rural communities, illness may be understood through relationships among the body, family, community, territory, and the spiritual world [60,61].

For some patients, end-of-life care also involves preparing the spirit, maintaining ties to the land, and honoring ancestral practices, which may conflict with conventional hospital protocols [62]. Although barriers such as the Kichwa language, ethnomedicine, and refusal of conventional treatments can generate moral distress, they also promote more respectful, flexible, and non-imposing clinical practice. These findings suggest that intercultural competence, including collaboration with community mediators, spiritual leaders, or cultural references, could be further explored as a potential area for future development in line with ALCP standards [4].

This need is consistent with evidence from community palliative care, where staff recognize the importance of cultural, religious, and spiritual needs but report limited confidence in addressing them within existing organizational structures. Strengthening proactive assessment, avoiding assumptions about patient beliefs, and providing practical tools, experience-based training, and accessible guidance may support more culturally and spiritually sensitive care [63].

Finally, in response to systemic barriers, interdisciplinary work has emerged as the main compensatory mechanism. Healthcare teams have developed coordinated interventions with high biographical meaning, such as weddings, sacraments, and legacy activities, despite limited structured training in spiritual communication [64]. This collective response may be explained by the familism and relational values characteristic of Latin America [8], which enable teams to act not only clinically but also as facilitators of existential closure.

Overall, this study highlights a gap between ALCP competency standards and the experiences described by the professionals interviewed. Within the Ecuadorian palliative care context represented in this sample, spiritual care relied on compassionate intuition and team resilience. These findings suggest that institutions and academic programs could strengthen spiritual literacy, intercultural mediation, and formal self-care strategies, particularly in contexts like those represented in this study.

These findings complement the previous literature by showing that, within the Ecuadorian palliative care context represented in this study, spiritual care is shaped not only by professional competencies but also by interactions among family decision-making, religious plurality, Indigenous worldviews, ancestral medicine, language barriers, and limited institutional structures.

### 4.4. Limitations and Opportunities of the Study

Despite the richness of the findings, this study has methodological limitations. Although the sample reached data saturation, it was predominantly composed of women, physicians, and urban residents, mainly from Quito. This composition may have influenced which experiences, barriers, and training needs were most strongly represented in the findings. Therefore, interpret the results mainly as reflecting the perspectives of ASECUP professionals who participated in the study, especially physicians working in urban palliative care settings, rather than the full interdisciplinary and territorial diversity of spiritual care in Ecuador.

In particular, nurses and psychologists were represented in smaller numbers than physicians. Since these professions may relate to spiritual care differently within interdisciplinary teams, this imbalance may have shaped which perspectives were most prominent in the findings.

In addition, other disciplines central to interdisciplinary palliative care, such as social workers, chaplains or pastoral care providers, community health workers, and traditional healers, were not represented in the final sample. Consequently, the findings may reflect mainly the perspectives of physicians and other clinical professionals rather than the full interdisciplinary community, pastoral, and ancestral reality of spiritual care.

Additionally, recruitment through ASECUP may have included professionals with greater sensitivity to existential suffering than the general healthcare workforce. As in qualitative research, findings should be interpreted within their specific context and participant characteristics, limiting transferability to other clinical or sociocultural settings [65].

These limitations also create opportunities for future research and practice development. Further studies could include patients, families, social workers, pastoral care providers, community health workers, and traditional healers to deepen understanding of total pain, spiritual suffering, and family involvement in decision-making. At the institutional level, future intervention studies could explore designing, implementing, and evaluating spiritual literacy curricula in health professions education. In addition, future research could examine ethically sensitive tools for documenting spiritual care in medical records. These areas should be understood as directions for future development rather than direct policy implications based on the present data.

## 5. Conclusions

Among the professionals interviewed, their responses aligned with the three ALCP competencies examined in this study: personal, spiritual, and professional development; ethics of spiritual care; and empathetic and compassionate communication. Participants drew on personal vulnerability, relational-ethical mediation, and nonverbal presence to provide spiritual accompaniment.

Together, these patterns define “compassionate intuition” as an unstructured, self-taught mode of spiritual care that emerged in this sample amid limited formal training and institutional protocols.

From a scientific standpoint, this study contributes to understanding how the professionals interviewed perceive and apply spiritual care within the Ecuadorian palliative care context represented by this sample. The findings suggest that spiritual care is often sustained through compassionate intuition, clinical sensitivity, personal vulnerability, and informal team support, rather than through fully structured institutional processes. Figure A1 summarizes this interpretive transition from compassionate intuition toward structured clinical competence.

From a practical standpoint, these findings point to possible areas for future development, including spiritual literacy in health professional education, clearer and ethically sensitive documentation processes, staff self-care and team support, and culturally sensitive mediation strategies in diverse care settings. These areas should be interpreted within the scope of this qualitative sample and examined in broader, more diverse contexts before being translated into institutional or national policies.

## Figures and Tables

**Table 1:** Semi-structured interview guide according to ALCP spiritual care competencies.

ALCP Competency	Item Assessed/Observable Behavior	Related Interview Questions
Sociodemographic and professional data	Description of participants’ personal, academic, professional, and training background.	Gender, age, nationality, country of residence, profession, level of education, years of healthcare experience, current healthcare setting, and training in palliative care and spiritual care.
Personal, spiritual, and professional development	Understanding of spirituality, religion, spiritual suffering, self-care, and quality spiritual care.	How do you understand your own spirituality? What type of knowledge or evidence do you consider necessary regarding spiritual care in your work context? How do you adapt this knowledge to daily clinical practice?
	Recognition of the role of one’s own spirituality, beliefs, and personal experiences in professional practice.	Can you describe an experience in which your personal or spiritual growth influenced the way you accompanied patients? How do your beliefs, personal experiences, and self-care practices influence the way you provide spiritual care?
	Development of self-awareness, self-reflection, and compassionate presence.	What activities, spaces, or people support your spirituality, compassionate presence, and personal or professional development, inside or outside the workplace?
	Recognition of burnout, compassion fatigue, and emotional exhaustion.	Have you experienced or identified emotional exhaustion in yourself or in your team? How did you manage it, and what strategies did you use?
	Continuous education, team learning, and collaborative support in spiritual care.	What training or learning experiences related to spiritual care have you shared with your team? How have these experiences strengthened the team’s ability to respond to patients’ spiritual needs?
	Participation in research or quality improvement related to spiritual care.	Have you had the opportunity to participate in or propose quality improvement or research projects related to spiritual care? How was that experience?
Ethics of spiritual care	Person-centered spiritual care, respect for dignity, and non-imposition of beliefs.	What does ethical and person-centered spiritual care mean to you? How is this reflected in your professional practice?
	Self-care is an ethical requirement for holistic and high-quality care.	How do you understand the importance of self-care in your professional practice? How does self-care influence your ability to provide comprehensive and high-quality spiritual care?
	Shared care planning with patients and caregivers, respecting values, beliefs, and decisions.	How do you plan care with patients and caregivers in order to respect their values, beliefs, and decisions? Can you describe an experience in which this represented an ethical challenge?
	Recognition and management of ethical dilemmas related to spiritual or religious needs at the end of life.	Have you encountered ethical issues related to the spiritual or religious dimensions of patients? How did you manage them, and what institutional support did you use?
	Identification of situations that may affect dignity or rights in relation to spiritual care.	Have you observed situations in your workplace that affected patients’ dignity or rights regarding spiritual issues? How did you respond, or what actions did you consider appropriate?
Empathic and compassionate communication	Active listening, empathic presence, and assertive, compassionate communication with patients and caregivers.	How would you describe your communication with patients and caregivers when spiritual issues arise? What experiences have helped you develop empathic listening?
	Documentation of relevant spiritual information, spiritual history, and care planning while respecting confidentiality.	How is patients’ spiritual information documented in clinical records or care planning? What importance do you attribute to documenting patients’ spiritual aspects during care?
	Identification of cultural, social, and religious barriers that hinder communication.	What cultural, social, or religious barriers have you encountered when discussing spiritual issues with patients or families? How have you addressed or overcome them?
	Development and sharing of spiritual care plans within the interdisciplinary team.	How do you develop and share spiritual care plans with your team, including assessment, interventions, and goals? Can you describe an experience in which team coordination was relevant?

**Table 2:** Sociodemographic and educational characteristics of the participants (n = 20).

Variable	Category	n	%
Profession	Physicians	14	70%
	Nurses	4	20%
	Psychologists	2	10%
Sex	Female	18	90%
	Male	2	10%
Place of Residence	Quito	11	55%
	Loja	4	20%
	Guayaquil	3	15%
	Machala	1	5%
	Riobamba	1	5%
Postgraduate Education	Yes	18	90%
	No	2	10%
Type of Palliative Care Training	Specialization	6	30%
	Master’s in Palliative Care	12	60%
	Diploma in Palliative Care	2	10%
Training in Spirituality	Diploma	2	10%
	No Training	18	90%
Employment Sector	Public	14	70%
	Private	6	30%
Level of Care	Primary	5	25%
	Secondary	11	55%
	Tertiary	4	20%

Table 2: Data are expressed as absolute frequencies (n) and percentages (%). The variable “spiritual training” refers exclusively to structured formal training. The level of care corresponds to the Ecuadorian health system’s classification. CP: Palliative care.

**Table 3:** Themes, subthemes, and representative quotations from the thematic analysis.

Theme	Subtheme	Representative Quotation
Personal, spiritual, and professional development	Self-care and emotional availability	“If you’re not well, you can’t take care of others; you have to be consistent” (C2, female, 61, nurse).
Personal, spiritual, and professional development	Clinical presence and self-awareness	“It has a huge impact… I have to be whole to be able to help them” (C15, female, 53, physician).
Ethics of spiritual care	Respect for spiritual expression	“Let be, let each individual be free to express… their spirituality in the way they conceive it” (C19, male, 52, physician).
Empathic and compassionate communication	Nonverbal and experiential communication	“Perceive, feel, listen, and also read the nonverbal aspects of the patients” (C18, female, 38, psychologist).
Empathic and compassionate communication	Creative communication strategies	“Death on the Table” (C18, female, 38, psychologist).
Interdisciplinary support	Informal team coordination	“This was done by word of mouth… during rounds” (C8, female, 30, psychologist).
Interdisciplinary support	Emotional support among professionals	“We shared our feelings… putting ourselves in each other’s shoes has helped us tremendously” (C18, female, 38, psychologist).
Institutional barriers	Lack of documentation tools	“There isn’t a specific section… it’s implicit, but not specific” (C10, female, 46, physician).
Institutional barriers	Confidentiality in documentation	“I never specify what those aspects are… it’s a very general reference” (C7, female, 60, physician).
Institutional barriers	Lack of time	“Patient care time [was] 20 min” (C20, female, 41, physician).
Institutional barriers	Need for time to build spiritual connection	“They systematize us a lot and don’t allow us time […] to have those spiritual connections we need to have time” (C19, male, 52, physician).
Sociocultural barriers	Language barriers	“I have many Kichwa-speaking patients… language is also something that is sorely lacking” (C9, female, 38, physician).
Sociocultural barriers	Traditional and ancestral medicine	“I don’t want chemotherapy… I’m going with my medicine, the natural and ancestral kind” (C9, female, 38, physician).
Sociocultural barriers	Confusion between spirituality and religiosity	“They confuse spirituality with religiosity, so that’s also a limitation” (C1, female, 34, physician).
Sociocultural barriers	Death as taboo	“The main one is that taboo… about death” (C15, female, 53, physician).
Ethical and family barriers	Family control over patient decisions	“Family members don’t want the patient’s decision respected; they want things done their way” (C1, female, 34, physician).
Ethical and family barriers	Dogmatic or religious barriers	“I don’t want a psychologist because my God is my only option” (C8, female, 30, psychologist).
Ethical and family barriers	Use of external mediators	“As a team, we had a consultation with a leader from that specific community [Jehovah’s Witnesses]” (C18, female, 38, psychologist).

**Figure A1:**
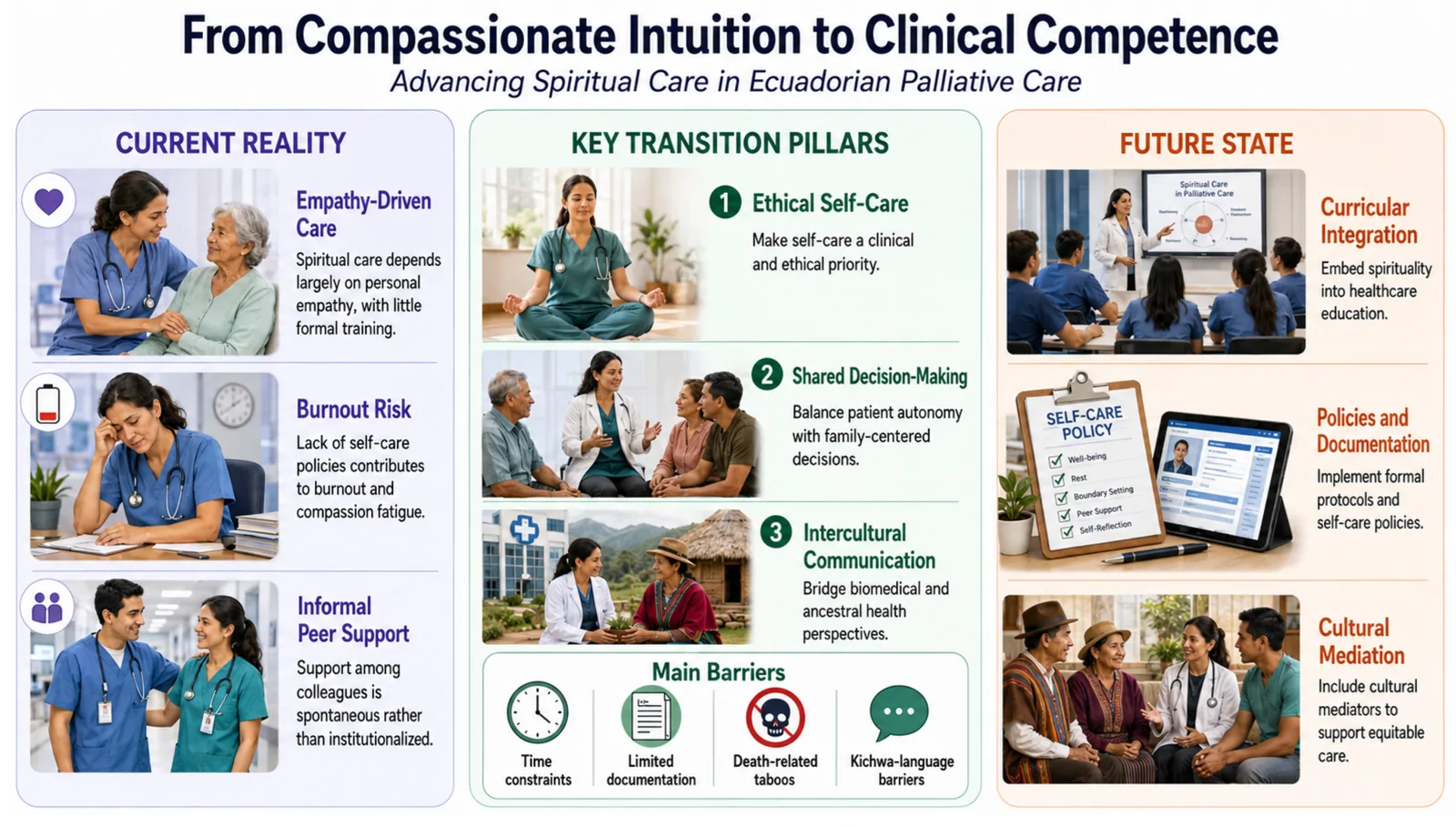
Interpretive model of compassionate intuition and structured clinical competence in palliative spiritual care.

## Data Availability

The data supporting this study’s findings are available from the corresponding author upon reasonable request.

## Supplementary Materials

The following supporting information can be downloaded at: https://www.mdpi.com/article/10.3390/healthcare14193197/s1, Table S1: Analytical audit trail from meaning units to initial codes, analytical categories, and final themes.

## Abbreviations

The following abbreviations are used in this manuscript:

ALCP	Latin American Association of Palliative Care
ASECUP	Ecuadorian Association of Palliative Care
