# Supplementary material for: From Compassionate Intuition to Clinical Competence: Spiritual Care Among Palliative Care Professionals in Ecuador
Source: Healthcare (Basel). 2026 Sep 28;14(19):3197. doi: 10.3390/healthcare14193197 (PMC13665089; doi:10.3390/healthcare14193197)
Supplement: Supplementary file 1 [file healthcare-14-03197-s001.zip › healthcare-4447597-supplementary.pdf]

Supplementary Table S1. Analytical audit trail from meaning units to initial codes, analytical categories, and final themes.

**Dataset / manuscript code**	**Anonymized meaning unit from the dataset**	**Initial code**	**Analytical category in the dataset**	**Final theme / interpretive output**
P007 / C7	Personal illness experience changed the way the professional accompanied patients clinically.	Personal vulnerability as a source of empathy	Personal vulnerability and empathy	Personal, spiritual, and professional development
P002 / C2	The professional recognized the need to be ethically prepared and personally able to accompany others.	Self-care as an ethical condition for care	Self-care as an ethical requirement	Ethics of spiritual care
P018 / C18	The professional emphasized reading patients' nonverbal expressions and remaining open to the clinical encounter.	Nonverbal presence	Nonverbal presence	Empathic and compassionate communication
P020 / C20	Administrative workload and institutional demands were described as sources of professional exhaustion.	Administrative burden	Sources of exhaustion	Institutional barriers
P002 / C2	The participant described persistent confusion between spirituality and religiosity as a limitation in care.	Spirituality–religiosity confusion	Conceptual barriers	Sociocultural barriers
P001 / C1	Family members were described as sometimes interfering with respect for the patient's decisions.	Family interference in decision-making	Autonomy, family, and decisions	Ethical and family barriers
P003 / C3	Informal conversations with colleagues helped professionals process emotionally difficult clinical situations.	Informal team containment	Team support and need for spiritual care specialist	Interdisciplinary support
Cross-case interpretive synthesis	Recurrent patterns included personal vulnerability, nonverbal presence, ethical-family mediation, informal team support, and limited formal training or documentation.	Relational and experiential response to spiritual suffering	Unstructured spiritual care practice	Interpretive construct: compassionate intuition

Note: The table presents representative examples from the anonymized dataset. It does not include the complete coding matrix. Dataset codes are shown together with manuscript quotation codes when applicable to facilitate traceability between the supplementary material and the revised manuscript.
